# Iselin Disease With a Rare Presentation in an Elderly Patient: A Case Report

**DOI:** 10.7759/cureus.33922

**Published:** 2023-01-18

**Authors:** Nibras K Aljabri, Turki H Alhassani, Atif A Labban, Abdullah H Alsawaf, Khalid A Alnofeay

**Affiliations:** 1 Orthopaedic Surgery, Al-Noor Specialist Hospital, Makkah, SAU; 2 Medical School, Umm Al-Qura University, Makkah, SAU; 3 Orthopaedic Surgery, King Faisal Hospital, Makkah, SAU

**Keywords:** elderly onset, ortho surgery, foot injuries, traction apophysitis, iselin’s disease

## Abstract

We report the case of a 55-year-old female with a rare presentation and different pathophysiology of Iselin’s disease. Iselin’s disease is a rare condition caused by traction apophysitis of the peroneus brevis tendon at the base of the fifth metatarsal bone. It is usually a case in the adolescent age group due to their repetitive use of the peroneus tendon in daily activities, but not in the elderly group.

This type of apophysitis is rare and easily missed or misdiagnosed as a fracture in the base of the fifth metatarsal bone. However, a simple radiographic image can confirm the diagnosis. In this case, we aim to raise awareness of Iselin’s disease for better comprehension of the clinical presentation, differential diagnosis, radiological features, management, and prognosis of Iselin’s disease.

## Introduction

Iselin’s disease is a benign, painful traction apophysitis or osteochondrosis, a cartilaginous prominence adjacent to the physis or growth plate. If this separates from the remainder of the bone and ossifies, it may mature and unite with the bone to form the protrusion of bone to which tendons are attached. This procedure occurs in Iselin's illness at the location where the peroneus brevis tendon inserts on the fifth metatarsal base. The fifth metatarsal apophysis is orientated longitudinally and serves as the attachment of the peroneus brevis tendon before skeletal maturity [1].

Iselin’s disease most commonly occurs in adolescents, affecting both males and females, first appearing on radiographs at approximately 8 to 11 years of age [1]. It is often confused with a fracture of the fifth metatarsal base when a patient presents with symptomatic fifth metatarsal base pain and undergoes subsequent radiography [2]. In this article, we detail the case of a 55-year-old female who is presented to our emergency department with other contributing factors that may affect disease management and progression.

## Case presentation

A 55-year-old female patient, a known case of diabetes mellitus for 25 years and hypertension for three years on medications, presented to the emergency department complaining of foot swelling on the lateral aspect of the right foot for one week. The swelling started gradually in the right foot at the plantar area and increased in size associated with pain during walking, throbbing in nature, not radiating anywhere. The pain was associated with redness, hotness, pus discharge, and generalized fever. The patient denied a history of trauma.

In her past medical history, the patient had a foreign body (plastic) for 15 years over her right foot plantar aspect; the patient did not seek medical attention then. She got pregnant; during pregnancy, the patient complained of fever and swelling over the plantar aspect of the right food. The patient was admitted to the hospital and underwent bedside irrigation and debridement, several rounds of incisions and drainage in the operation room, and stayed in the hospital for three months. After that, she was referred to another hospital for a skin graft.

**Figure 1:**
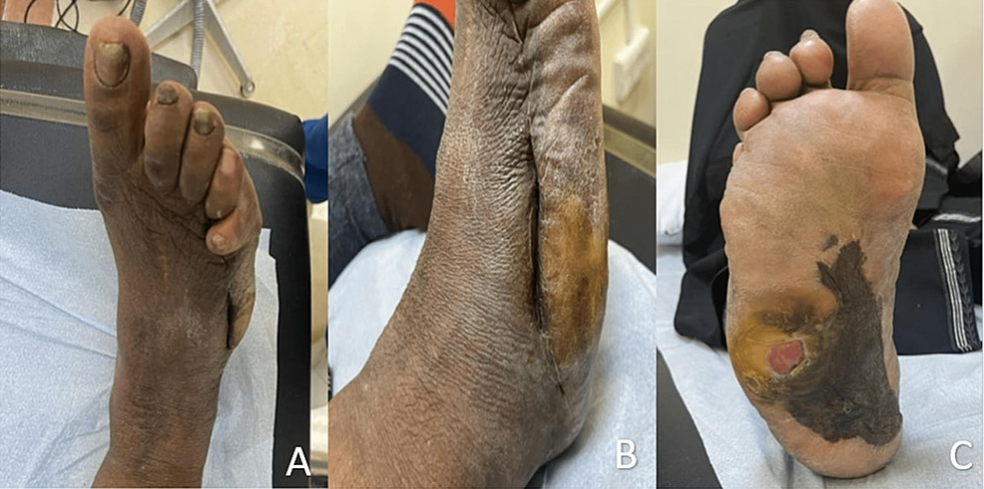
(A) The dorsum aspect of the foot and deformed fifth toe with a lateral scar. (B) The lateral aspect of the foot with a scar extending from the fifth toe up to the heel. (C) The plantar aspect of the foot and ulcer at the lateral side with a plastic foreign body covered medial lower side.

On examination, there was swelling at the right foot in the planter area with a collection of about 2x3 cm, negative fluctuation, associated with hotness, redness, tenderness, and pitting edema on the dorsum aspect of the foot, and no pus or blood discharge. There was an unhealed ulcer in the lateral aspect of the sole. Her neurovascular examination was normal (Figure 1).

**Figure 2:**
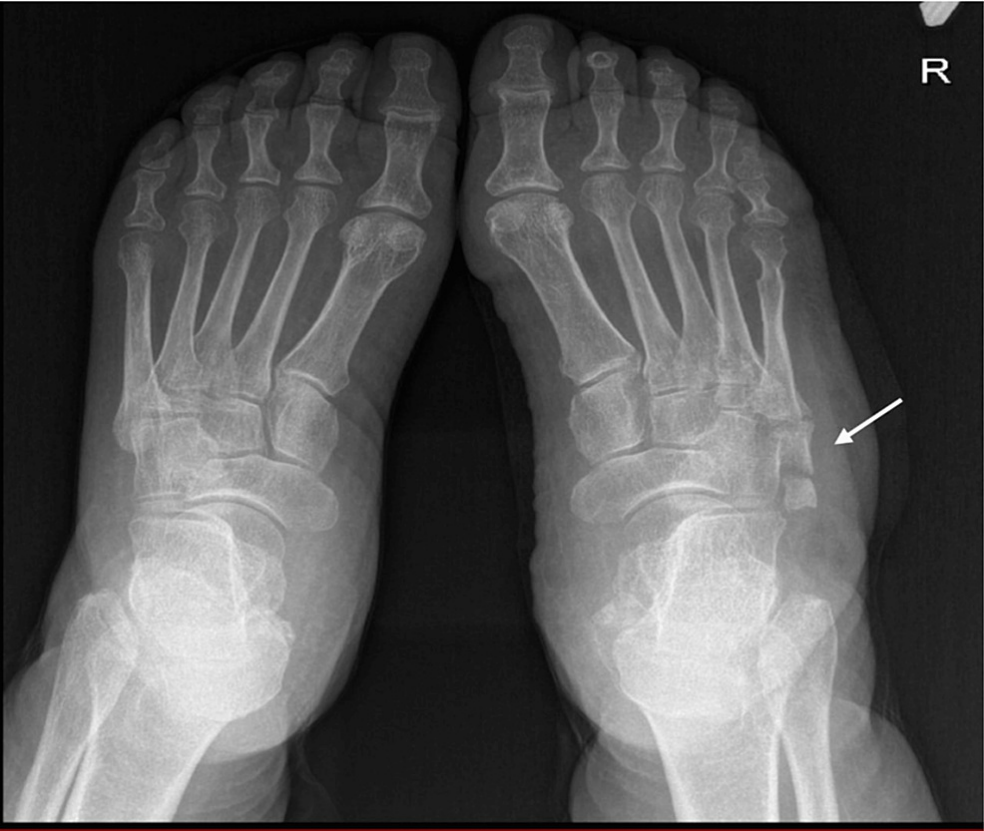
Bilateral foot X-ray anteroposterior view - accessory bone in the base of the right fifth metatarsal bone (arrow).

**Figure 3:**
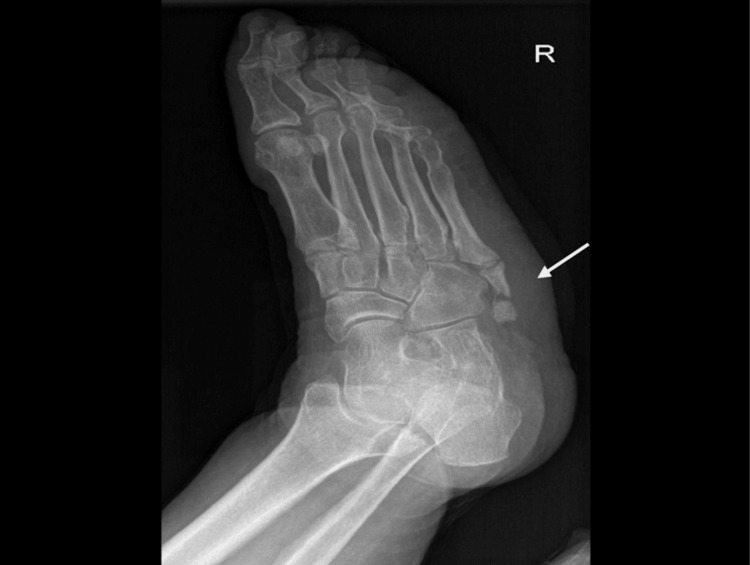
Right foot X-ray oblique view - accessory bone connected to the base of the fifth metatarsal bone.

On radiological evaluation, the right foot X-ray revealed an accessory bone growth at the base of the fifth metatarsal bone (Figures 2, 3). Ultrasound was requested for to rule out deep tissue collection; the report was suggestive of a collection measuring about 3x1 cm in maximum diameter on the lateral aspect of the right foot, with no internal vascularity, and diffuse subcutaneous edema in the dorsum aspect of the right foot.

**Figure 4:**
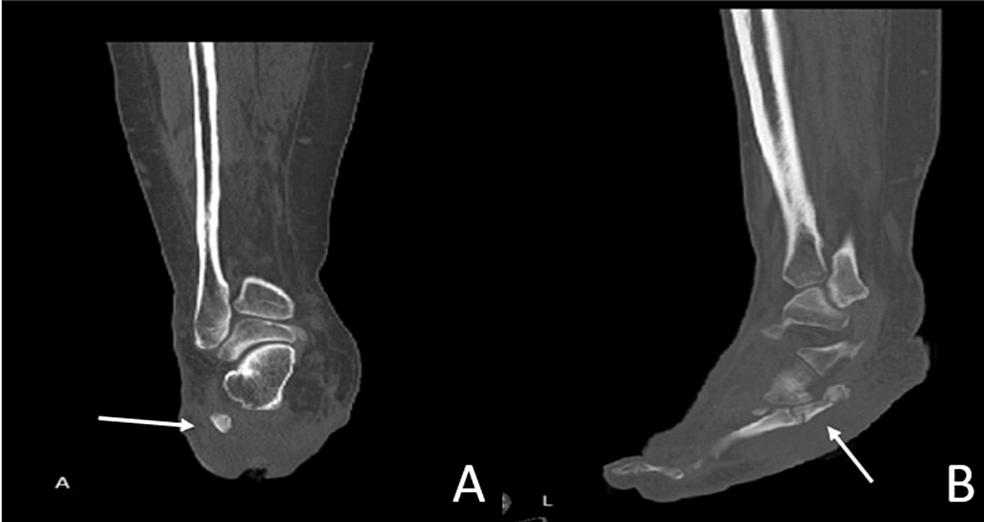
CT scan of the lower foot. (A) Coronal view of the right tarsal bones with an accessory bone. (B) Sagittal view of the right foot with an accessory bone connected to the base of the fifth metatarsal bone.

A CT scan was done on the patient. It showed evidence of an accessory bone seen posterior to the proximal fifth metatarsal base with evidence of articulation point to the cuboid bone. The fracture line was seen traversing this accessory bone (Figure 4).

The patient was discharged on cefuroxime 500 mg PO q12hr for ten days with daily dressing for ulcer management and paracetamol 1 g per q8h if needed. She was advised not to bear weight and scheduled for MRI to look for any signs of osteomyelitis. After one month, the patient came for a follow-up in the clinic. She was pain-free and able to walk on her feet and manage her daily activities. An MRI shows no signs of osteomyelitis.

## Discussion

The first description of Iselin disease was traction apophysitis of the fifth metatarsal base in 1912. Although the precise cause of Iselin’s disease is unknown, when it affects adolescents, it is referred to as osteochondrosis [1]. The friction of the peroneus brevis tendon at the insertion point turns an additional site of ossification from the body of the fifth metatarsal. Thus, the apophysis will experience repetitive micro traumata due to the strain experienced during athletic activity. Adolescents who participate in sports requiring running and leaping develop this condition. Adolescents who participated in gymnastics, dance, roller skating, soccer, basketball, and athletics were among the reported cases [1-3]. However, our patient was a 55-year-old female patient with no history of trauma or repetitive motion that could be linked to the etiology of this disease.

Symptoms of Iselin disease include pain across the lateral midfoot. Resting helps to ease the pain, which gradually develops and is exacerbated by activities like jogging and leaping. Intermittent limping and swelling over the lateral midfoot may also be presenting symptoms. Upon inspection, the tuberosity may expand and soft tissue may bulge. The most prominent clinical symptoms include discomfort when walking or hopping and painful palpation over the fifth metatarsal tuberosity [4]. Edema, erythema, and ecchymosis are typically extremely minimal or non-existent. Both plantar flexion and resisted eversion are painful. The doctor should look for instability, deformity, joint crepitus, and loss of mobility during the clinical examination [1].

Radiographic examination is critical in the diagnosis of Iselin's disease. To obtain a diagnosis of Iselin's disease, radiographic results must be matched with clinical symptoms. Other forms of imaging are not needed to make the diagnosis [5]. Other modalities can be used to rule out other differential diagnoses. After ruling out other probable causes of discomfort and swelling around the fifth metatarsal base, Iselin's disease should be diagnosed. Jones fracture, avulsion fracture, stress fracture at the base of the fifth metatarsal, and os vesalianum are all possible diagnoses. An accurate history is needed to rule out the history of previous trauma, penetrating injury, and infection. Apophysitis is still a clinical diagnosis backed by radiological studies.

The majority of the management of Iselin disease is conservative. The initial treatment focuses on removing the causative factors. In mild cases, wrapping ice packs across the foot's lateral part helps reduce inflammation. In the past, non-steroidal anti-inflammatory drugs (NSAIDs) were recommended to reduce inflammation [2,6]. It is critical to advise your patient to avoid sports and other activities that cause discomfort. This implies no sports for a few weeks, followed by partial weight-bearing crutches. Physical treatment with exercises to strengthen the peroneus muscles, enhance the range of motion, and improve flexibility should be started after immobilization when the discomfort and tenderness have subsided [7].

## Conclusions

Iselin disease is a self-limiting condition that often improves with conservative management. It has a favorable prognosis. When young athletes are pain-free and have regained their full strength, they may resume their sport. To avoid recurrence, radiographic evaluation to determine whether the teenager is ready to resume athletics is helpful. Typically, in Iselin disease, it takes 3 to 12 weeks for the pain to subside. Physicians should focus on management failure, nevertheless. When Iselin disease is not adequately managed, non-union might occur. Surgical intervention may be necessary, either by excision or fixation at the non-union site. As long as the removed bone does not interfere with the peroneus brevis tendon's functionality or the gait stability, excision of the proximal osseous fragment is advised.
